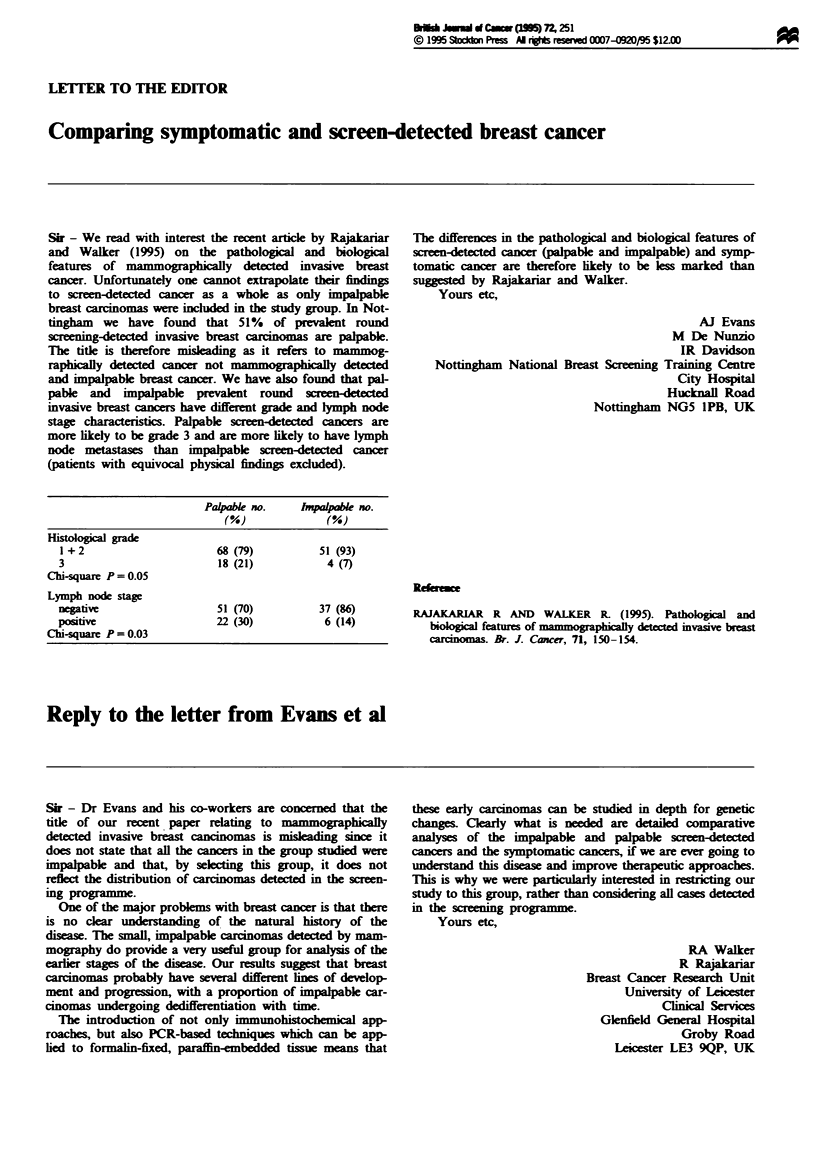# Comparing symptomatic and screen-detected breast cancer.

**DOI:** 10.1038/bjc.1995.313

**Published:** 1995-07

**Authors:** A. J. Evans, M. De Nunzio, I. R. Davidson


© 1995 Stockton Press All rights reserved 0007-0920/95 $12.00

LETTER TO THE EDITOR

### Comparing symptomatic and screen-detected breast cancer

Sir – We read with interest the recent article by Rajakariar and Walker (1995) on the pathological and biological features of mammographically detected invasive breast cancer. Unfortunately one cannot extrapolate their findings to screen-detected cancer as a whole as only impalpable breast carcinomas were included in the study group. In Nottingham we have found that 51% of prevalent round screening-detected invasive breast carcinomas are palpable. The title is therefore misleading as it refers to mammographically detected cancer not mammographically detected and impalpable breast cancer. We have also found that palpable and impalpable prevalent round screen-detected invasive breast cancers have different grade and lymph node stage characteristics. Palpable screen-detected cancers are more likely to be grade 3 and are more likely to have lymph node metastases than impalpable screen-detected cancer (patients with equivocal physical findings excluded).

| | *Palpable no. (%)* | *Impalpable no. (%)* |
|---|---|---|
| Histological grade | | |
| 1 + 2 | 68 (79) | 51 (93) |
| 3 | 18 (21) | 4 (7) |
| Chi-square $P = 0.05$ | | |
| Lymph node stage | | |
| negative | 51 (70) | 37 (86) |
| positive | 22 (30) | 6 (14) |
| Chi-square $P = 0.03$ | | |

The differences in the pathological and biological features of screen-detected cancer (palpable and impalpable) and symptomatic cancer are therefore likely to be less marked than suggested by Rajakariar and Walker.

Yours etc,

AJ Evans
M De Nunzio
IR Davidson
Nottingham National Breast Screening Training Centre
City Hospital
Hucknall Road
Nottingham NG5 1PB, UK

### Reference

RAJAKARIAR R AND WALKER R. (1995). Pathological and biological features of mammographically detected invasive breast carcinomas. *Br. J. Cancer*, **71**, 150-154.